# Fatal Postoperative Interstitial Lung Disease after Neoadjuvant Nivolumab and Complete Resection for Non-Small Cell Lung Cancer: A Case Report

**DOI:** 10.70352/scrj.cr.25-0363

**Published:** 2026-04-01

**Authors:** Takatoshi Osako, Aki Shimamura, Teruhisa Takuwa

**Affiliations:** Department of Thoracic Surgery, Saiseikai Noe Hospital, Osaka, Osaka, Osaka, Japan

**Keywords:** interstitial lung disease, nivolumab, neoadjuvant chemotherapy

## Abstract

**INTRODUCTION:**

Neoadjuvant immunotherapy with nivolumab combined with chemotherapy has become a standard treatment for resectable non-small cell lung cancer (NSCLC), demonstrating improved survival outcomes. However, immune checkpoint inhibitors (ICIs) such as nivolumab are associated with immune-related adverse events, including interstitial lung disease (ILD). While ILD is a recognized complication in advanced NSCLC, its occurrence following neoadjuvant ICI therapy and subsequent surgical resection is rarely reported.

**CASE PRESENTATION:**

An 84-year-old man with stage IIB squamous cell carcinoma of the left lung received three cycles of neoadjuvant chemotherapy with nivolumab, carboplatin, and paclitaxel, followed by left upper lobectomy and mediastinal lymph node dissection. The initial postoperative course was uneventful; however, on POD 7, the patient developed elevated inflammatory markers and radiologic findings suggestive of interstitial changes. Despite the administration of corticosteroids and antibiotics, his condition deteriorated, leading to respiratory failure and death on POD 25. Imaging revealed a diffuse alveolar damage pattern, consistent with severe ILD.

**CONCLUSIONS:**

This case highlights the potential risk of fatal ILD following neoadjuvant nivolumab therapy and surgical resection in NSCLC patients. Clinicians should maintain a high index of suspicion for ILD in the perioperative period, even in patients without pre-existing pulmonary disease, and consider early diagnostic imaging and timely corticosteroids therapy when appropriate.

## Abbreviations

ARDS
: acute respiratory distress syndrome
CRP
: C-reactive protein
DAD
: diffuse alveolar damage
GGO
: ground-glass opacities
ICI
: immune checkpoint inhibitor
ILD
: interstitial lung disease
TIA
: transient ischemic attack

## INTRODUCTION

In the CheckMate 816 trial (ClinicalTrials.gov number, NCT02998528.), neoadjuvant nivolumab combined with chemotherapy significantly improved event-free survival and the pathological complete response rate when compared with chemotherapy alone.^1)^ As a result, nivolumab plus chemotherapy has become a standard approach for neoadjuvant treatment in resectable non-small cell lung cancer. In this trial, only two cases of surgery-related grade 5 complications were reported, and both were deemed unrelated to the trial drugs.^1)^ However, we present a case in which a patient who underwent lung resection after receiving neoadjuvant nivolumab plus chemotherapy developed interstitial lung disease (ILD) and ultimately died. Although the relationship between nivolumab and the development of ILD in this case cannot be definitively established, it highlights the need for heightened awareness of this potential complication. This case underscores the importance of closely monitoring for ILD in patients receiving nivolumab as part of neoadjuvant treatment regimens.

## CASE PRESENTATION

**Fig. 1:**
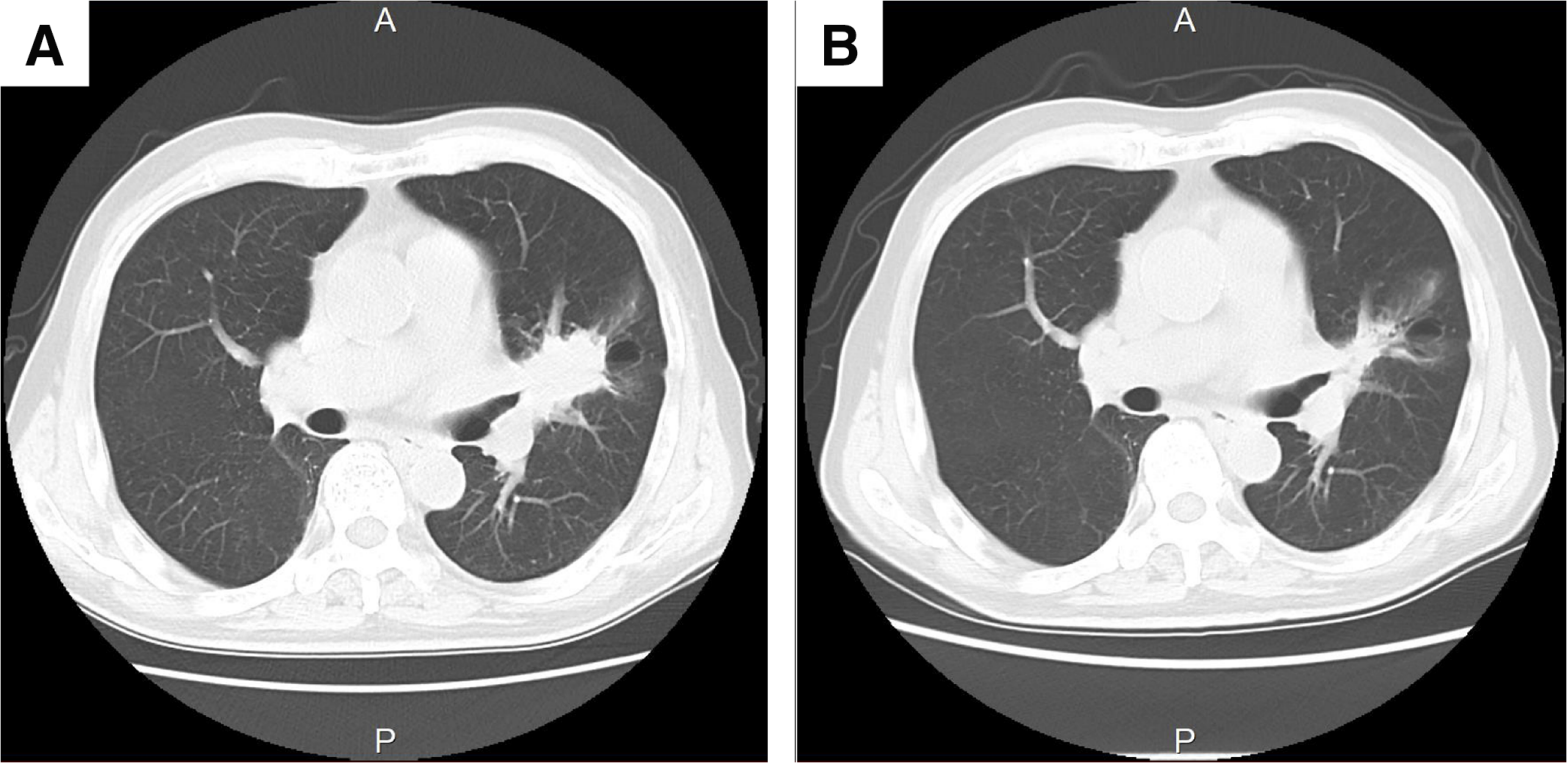
CT images obtained (**A**) before and (**B**) after neoadjuvant chemotherapy. The tumor decreased in size following chemotherapy, and no shadows suggestive of ILD were observed before or after treatment.

An 84-year-old male presented with an elevated carcinoembryonic antigen level during routine blood tests. Chest CT revealed a 4.8 cm mass in the left pulmonary hilar region, with pathological examination confirming squamous cell carcinoma of the left lung (**Fig. 1A**). PET-CT showed an accumulation with a maximum standardized uptake value of 33.5 in the area extending from the tumor to the hilar lymph nodes. The tumor was classified as clinical stage IIB (cT2bN1M0), with extensive involvement of the hilar lymph nodes but no evidence of distant metastasis. The patient’s medical history included hypertension and a transient ischemic attack (TIA), for which he was on antiplatelet therapy. He had no history of interstitial lung disease or pulmonary fibrosis. His smoking history consisted of 30 cigarettes per day until the age of 40. Preoperative pulmonary function tests were within normal limits, with a forced vital capacity of 3.10 L (103.7% predicted) and a forced expiratory volume in 1 second of 2.46 L (107.4% predicted).

ILD, interstitial lung disease

The patient underwent three cycles of neoadjuvant chemotherapy consisting of nivolumab (360 mg/body), carboplatin (AUC 5), and paclitaxel (175 mg/m^2^). During chemotherapy, the patient experienced grade 4 neutropenia but completed all cycles without dose reduction. Following chemotherapy, the tumor shrank to 3.2 cm, as assessed by CT imaging (**Fig. 1B**). Surgery was performed 6 weeks after chemotherapy, consisting of a left upper lobectomy and mediastinal lymph node dissection. The operation lasted 154 minutes, with a blood loss of 135 mL, and was completed without intraoperative complications.

**Fig. 2:**
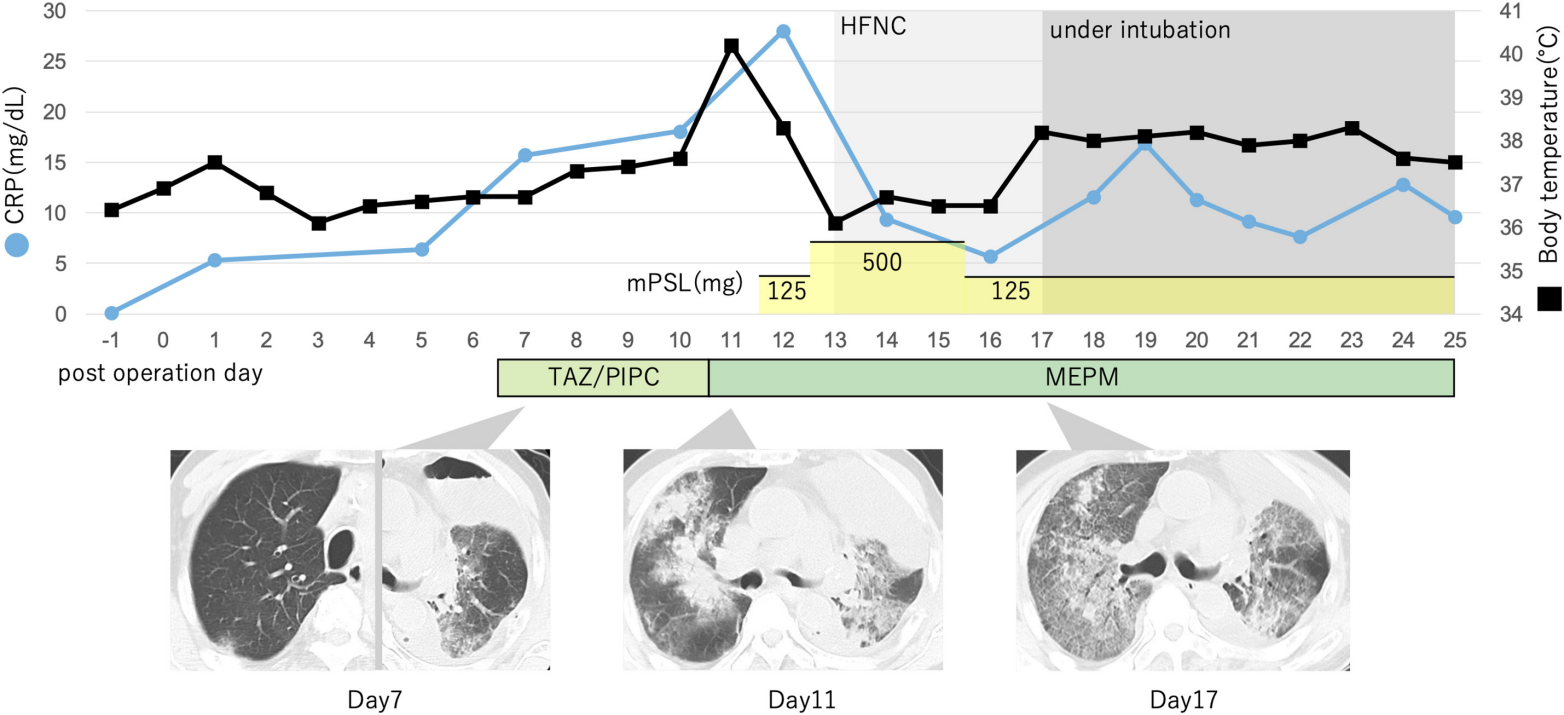
Timeline of the CT findings, body temperature, CRP level, and details of treatment, with the day of surgery designated as day 0. On POD 7, chest CT revealed bilateral ground-glass opacities, and blood tests showed an increase in CRP to 15.73 mg/dL. Although the patient was asymptomatic, treatment with the antibiotics TAZ and PIPC was initiated. On POD 11, the patient developed a fever, and chest CT showed an enlargement of the opacities. The antibiotics were switched to MEPM, and mPSL was also started. This led to improvements in body temperature and CRP levels, but the respiratory condition worsened. On POD 13, anHFNC was applied, and treatment with high-dose mPSL (500 mg/day) was initiated. However, the respiratory condition continued to deteriorate, and on POD 17, mechanical ventilation was initiated. At that point, chest CT showed a DAD pattern. The patient passed away on POD 25.

CRP, C-reactive protein; DAD, diffuse alveolar damage; HFNC, high-flow nasal cannula; MEPM, meropenem; mPSL, methylprednisolone; TAZ/PIPC, tazobactam and piperacillin

Postoperatively, the patient’s initial recovery was uneventful. He began walking independently and resumed oral intake on POD 1. Despite minor air leakage requiring thoracic drainage for 4 days, his progress remained smooth until POD 6. However, on POD 7, blood tests revealed leukocytosis (13500/μL) and an elevated C-reactive protein (CRP) level of 15.73 mg/dL. CT scans showed ground-glass opacities (GGO) in the left lung and subpleural regions of the right lung. Echocardiography showed no evidence of left ventricular dysfunction, ruling out cardiogenic pulmonary edema. Antibiotic therapy with tazobactam/piperacillin was initiated despite the absence of respiratory symptoms. On POD 11, the patient developed a high-grade fever (40°C), dyspnea, and progressive hypoxemia. Repeat CT scans revealed bilateral infiltration consistent with an ILD pattern. Meropenem was introduced, and 125 mg/day of methylprednisolone was added. Despite this, his respiratory status worsened, requiring high flow nasal cannula therapy on POD 13. High-dose methylprednisolone (500 mg/day) was administered for 3 days, but the patient’s condition deteriorated, necessitating mechanical ventilation. CT findings at this stage demonstrated a diffuse alveolar damage (DAD) pattern. The patient’s respiratory failure persisted despite aggressive treatment. He passed away on POD 25 (**Fig. 2**). Sputum and blood cultures obtained during treatment showed no bacterial growth, and the β-D-glucan level was not elevated. The clinical course suggested severe ILD, likely immune-related and associated with neoadjuvant nivolumab therapy.

## DISCUSSION

In this case, surgical biopsy and bronchoalveolar lavage could not be performed because of the progressive deterioration of the patient’s respiratory status. The diagnosis of ILD in this case was supported by the lack of response to broad-spectrum antibiotics, negative microbiological test results, postoperative findings of leukocytosis, elevated CRP, GGO on chest CT, and progressive respiratory failure with a DAD pattern observed on later CT scans. These findings were subtle and nonspecific in the early postoperative period. However, the absence of underlying diseases known to cause ILD, such as collagen vascular disease, and the fact that the patient had been treated with nivolumab were decisive factors in diagnosing drug-induced ILD. Nivolumab-associated ILD has been reported to occur in 3.5%–4.6% of cases in clinical trials.^2,3)^ Among Japanese patients, this rate is higher, ranging from 7.2% to 9.6%, with fatal cases also reported.^4,5)^ The median onset period is approximately 55 days (ranging from 1 to 365 days) after the start of treatment.^5)^ In this case, no shadows indicative of ILD were observed before treatment, and the onset occurred postoperatively. Although the onset was delayed, preoperative administration of nivolumab likely contributed to the development of ILD. The interval from the last chemotherapy administration to surgery was 6 weeks, and neutropenia had resolved before surgery. Therefore, we consider it unlikely that agents other than nivolumab had any effect on the postoperative course.

The interplay between surgical stress and nivolumab-induced immune activation cannot be ignored. Although the surgical procedure was uncomplicated, the trauma of lung resection and mediastinal lymph node dissection may have amplified systemic inflammation, potentially triggering immune dysregulation and accelerating the onset of ILD. Patients with ILD have a significantly higher postoperative mortality rate due to complications such as acute respiratory distress syndrome (ARDS) and pneumonia.^6)^ In this case, surgical stress combined with nivolumab-associated immune activation likely exacerbated pulmonary inflammation, leading to ARDS and the fatal outcome.

Notably, this patient was elderly with a history of hypertension and TIA, but had no prior history of pulmonary disease. This emphasizes that ILD risk exists even in patients without preexisting lung conditions. The patient’s former smoking history, while not a direct contraindication for ICI therapy, may have contributed to baseline lung vulnerability, particularly when coupled with surgical stress.

Early intervention is critical in the management of ILD. Antibiotics were promptly initiated following the detection of inflammatory markers and GGO, which was appropriate considering the differential diagnosis included postoperative infection. However, corticosteroids were introduced only after significant clinical deterioration. Closer monitoring of blood tests and radiologic findings was necessary to assess the effectiveness of antibiotic therapy in a timely manner. Earlier initiation of high-dose corticosteroid therapy, once it became evident that antibiotic treatment was ineffective, might have suppressed immune-mediated pulmonary inflammation more effectively. In retrospect, the response to antibiotics initiated on POD 7 should have been assessed for 2–3 days, and high-dose steroid pulse therapy should have been started from POD 9. These findings underscore the need for standardized approaches to the timing and thresholds for corticosteroid administration in suspected ILD following ICI therapy.

This case underscores the complexity of managing ILD in the perioperative setting, particularly in patients receiving ICIs. Future research should focus on developing predictive models for ILD risk stratification and optimizing perioperative care for patients undergoing surgery after ICI therapy.

## CONCLUSIONS

We experienced fatal postoperative ILD after neoadjuvant chemotherapy combined with nivolumab and complete resection for non-small cell lung cancer. When ICIs are used preoperatively, it is essential to carefully monitor the postoperative course with the potential risk of ILD in mind.

## DECLARATIONS

### Funding

No funding sources were received.

### Authors’ contributions

TO and AS analyzed and interpreted the patient data.

TT and TO drafted the manuscript.

All authors participated in the patient management and contributed to discussions on the intellectual content of the manuscript.

All authors read and approved the final manuscript.

### Availability of data and materials

Not applicable.

### Ethics approval and consent to participate

This work does not require ethical considerations or approval. Informed consent for this report was obtained from the patient’s family.

### Consent for publications

Consent for the publication was obtained from the patient’s family.

### Competing Interests

The authors declare that they have no competing interests.
